# The upper frequency limit for the use of phase locking to code temporal fine structure in humans: A compilation of viewpoints

**DOI:** 10.1016/j.heares.2019.03.011

**Published:** 2019-06

**Authors:** Eric Verschooten, Shihab Shamma, Andrew J. Oxenham, Brian C.J. Moore, Philip X. Joris, Michael G. Heinz, Christopher J. Plack

**Affiliations:** a Laboratory of Auditory Neurophysiology, KU Leuven, B-3000, Leuven, Belgium; b Institute for Systems Research and Electrical and Computer Engineering, University of Maryland, College Park, MD, 20742, USA; c Laboratory of Sensory Perception, Department of Cognitive Studies, Ecole Normale Superieure, 29 Rue d'Ulm, Paris, 75005, France; d Department of Psychology, University of Minnesota, N218 Elliott Hall, 75 E. River Road, Minneapolis, MN, 55455, USA; e Department of Psychology, University of Cambridge, Downing Street, Cambridge, CB2 3EB, UK; f Departments of Speech, Language, & Hearing Sciences and Biomedical Engineering, Purdue University, 715 Clinic Drive, West Lafayette, IN, 47907, USA; g Manchester Centre for Audiology and Deafness, The University of Manchester, Manchester Academic Health Science Centre, M13 9PL, UK; h Department of Psychology, Lancaster University, Lancaster, LA1 4YF, UK

**Keywords:** Phase locking, Temporal fine structure, Temporal coding, Place coding, Pitch

## Abstract

The relative importance of neural temporal and place coding in auditory perception is still a matter of much debate. The current article is a compilation of viewpoints from leading auditory psychophysicists and physiologists regarding the upper frequency limit for the use of neural phase locking to code temporal fine structure in humans. While phase locking is used for binaural processing up to about 1500 Hz, there is disagreement regarding the use of monaural phase-locking information at higher frequencies. Estimates of the general upper limit proposed by the contributors range from 1500 to 10000 Hz. The arguments depend on whether or not phase locking is needed to explain psychophysical discrimination performance at frequencies above 1500 Hz, and whether or not the phase-locked neural representation is sufficiently robust at these frequencies to provide useable information. The contributors suggest key experiments that may help to resolve this issue, and experimental findings that may cause them to change their minds. This issue is of crucial importance to our understanding of the neural basis of auditory perception in general, and of pitch perception in particular.

## Highlights

• Phase locking is used in binaural processing for frequencies up to ∼1500 Hz.
• Estimates of the general upper limit (inc. monaural processing) vary from 1500 to 10000 Hz.
• Direct recordings from human auditory nerve would determine peripheral limitation.
• Understanding of the central processing of temporal and place cues is needed to establish an upper limit.

## 1 Introduction

Temporal fine structure (TFS) refers to the individual pressure fluctuations in a sound waveform. After spectral decomposition in the cochlea, TFS information is present in the vibration of the basilar membrane at each characteristic frequency (CF), and is preserved to some extent by the synchronized firing patterns (phase locking) of neurons in the auditory nerve (Rose et al., 1967; Johnson, 1980) and at higher centers in the auditory pathway. TFS information is known to be important in sound localization, and there is evidence for a role for TFS information in pitch perception and in speech perception (Moore, 2008).

However, despite many decades of intensive research, there is still controversy regarding the relative importance of phase-locking information and place (or rate-place) information for coding sound frequency: To what extent is audio frequency coded by the phase-locked neural firing patterns or by the place on the basilar membrane or in the auditory nerve that is excited? A particular point of contention concerns the use of TFS information at high frequencies. Direct measurements of phase locking require the use of techniques currently not feasible in humans, and a comparative approach is necessary in order to understand the relations between physiology and perception. Animal experiments provide evidence that neural phase locking can represent TFS up to a certain frequency, but there is debate regarding what this frequency is in humans, and regarding the relative importance of phase locking and place information to the perception of sounds at high frequencies. This is of crucial importance to our understanding of how hearing works, and represents an important gap in our knowledge.

The spotlight has been thrown in particular on pitch perception and frequency discrimination. Many authors have assumed that musical pitch is dependent on a temporal code, in part because musical pitch perception seems to break down for frequencies above the limit of neural phase locking in animal models (about 5000 Hz: Attneave and Olson, 1971; Semal and Demany, 1990). However, the results of Oxenham and colleagues call this into question (Oxenham et al., 2011; Lau et al., 2017). Their evidence that complex tones consisting entirely of harmonics above 8000 Hz can produce a robust musical pitch percept, suggest that phase locking is not necessary for the perception of musical pitch. Is the pitch code then a “dual mechanism,” involving both place and temporal information (Cedolin and Delgutte, 2005)? Is it even possible that temporal information is of limited importance in pitch perception? Knowledge of the upper frequency limit of temporal coding will contribute greatly to our understanding of these processes.

In the present article, several authorities on pitch perception and neural temporal coding answer the following questions:

1. What is the highest frequency for which phase locking is used to code temporal fine structure in humans?
2. What key future experiments would help resolve the issue?
3. What experimental finding would cause you to change your mind?

Question 1 is simple to express, but requires consideration of a range of complex experimental evidence to answer. There are basic neurophysiological aspects, largely dependent on animal data, related to the ability of neurons to phase lock and to process phase-locking information effectively, and perceptual aspects related to the use of that information by the brain to make discriminations between sounds. It is not sufficient to show that the human auditory nerve can represent temporal information at a certain frequency; it is necessary to show that this information is used by the auditory brain. Question 2 is intended to produce responses that will illuminate the path for future research endeavor in this area, and in particular to encourage the next generation of researchers to get stuck in. Finally, Question 3 is intended to put the authors on the spot: for them to demonstrate that their theoretical position is falsifiable (and hence is science rather than dogma), and to commit them to changing their mind, should the results turn against them.

## 2 Brian Moore

### 2.1 What is the highest frequency for which phase locking is used to code temporal fine structure in humans?

My best estimate of this limit, about 8000 Hz, is probably at the high end of the range of estimates given in this paper. Let me give my reasons for that estimate. In what follows, I denote the TFS of sounds as represented in patterns of phase locking in the auditory nerve as TFS_n_, following Moore (2014).

The role of TFS_n_ information in the perception of the pitch of sinusoids has been inferred from the way that frequency discrimination changes with center frequency. If frequency discrimination depends on place information, then difference limens for frequency (DLFs) should depend on two factors: the sharpness of the excitation pattern evoked by a sinusoid and the smallest detectable change in excitation level (Zwicker, 1956). When expressed relative to the center frequency, excitation patterns are at least as sharp at high frequencies as they are at low frequencies (Glasberg and Moore, 1990; Oxenham and Shera, 2003). The smallest detectable change in excitation level is also approximately constant across a wide frequency range (Florentine, 1983), except for a worsening at medium sound levels for very high frequencies and short tones (Carlyon and Moore, 1984). Therefore, if frequency discrimination depends on place cues, DLFs for long-duration tones, when expressed as a proportion of the baseline frequency, should be approximately constant across medium and high frequencies. In contrast, phase locking weakens at high frequencies, so if DLFs increase at high frequencies, this provides evidence for a role of TFS_n_ information at lower frequencies.

Based on these ideas, Moore and Ernst (2012) measured DLFs over a wide frequency range, including frequencies well above 8000 Hz. The subjects were selected to have audiometric thresholds better than 20 dB HL for frequencies up to 14000 Hz. The task was designed to be easy to learn and not to require naming of the direction of a pitch change, which is difficult for some subjects (Semal and Demany, 2006). In one randomly selected interval of a trial, there were four successive 500-ms tone bursts with a fixed frequency, *f*. In the other interval, the frequency alternated between *f* and *f*+Δ*f*. The task of the subject was to choose the interval in which the sound changed across the four tone bursts within an interval. The value of Δ*f* was adaptively varied to determine the DLF. To reduce the availability of loudness cues, the stimuli were presented via earphones with a “flat” response at the eardrum and the level of every tone was varied randomly over a range of ±4 dB (uniform distribution) around the mean level. For each frequency, the DLF was estimated for a mean level of 70 dB SPL and for a mean sensation level (SL) of 20 dB.

**Fig. 1:**
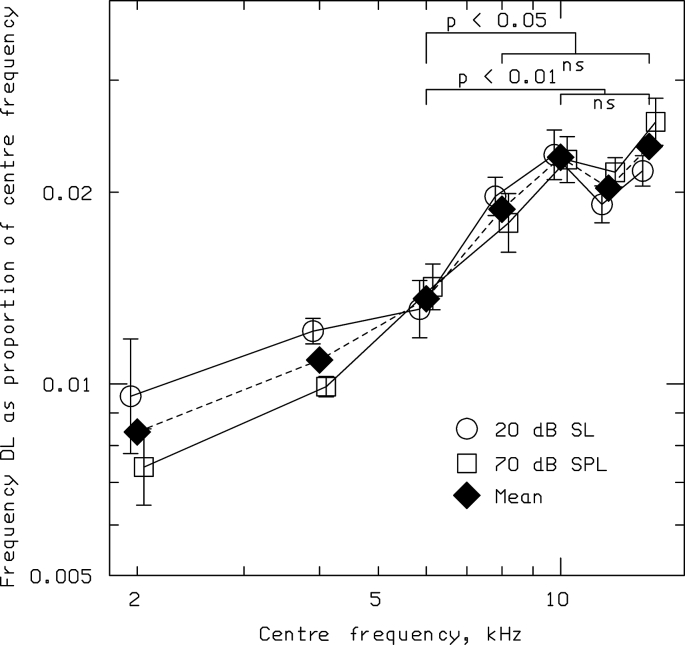
Data from Moore and Ernst (2012) showing geometric mean values of DLF/*f* across subjects, plotted as a function of *f*. Circles and squares show values obtained at 20 dB SL and 70 dB SPL, respectively. Diamonds show geometric means for the two levels. The outcomes of statistical tests of the differences across *f* are shown at the top.

Fig. 1 shows the geometric mean values of DLF/*f* across subjects. Circles and squares show values obtained at 20 dB SL and 70 dB SPL, respectively. Diamonds show geometric means for the two levels. DLF/*f* increased with increasing frequency up to 8000 or 10000 Hz and then flattened off. There was no significant difference between the values of DLF/*f* for center frequencies from 8000 to 14000 Hz, but the values of DLF/*f* for all these center frequencies were significantly greater than the value at 6000 Hz.

These results are consistent with the idea that DLFs depend on TFS_n_ information at low frequencies and place information at high frequencies. Over the frequency range where the place mechanism is dominant, DLF/*f* is roughly independent of *f*. The transition between the two mechanisms appears to occur at about 8000 Hz.

The role of TFS_n_ information in the perception of the pitch of complex tones has been studied using complex tones that contain many harmonics but are bandpass filtered so as to contain only high unresolved harmonics, above about the eighth (Moore and Gockel, 2011). A background noise is added to mask combination tones and to limit the audibility of components falling on the skirts of the bandpass filter (Moore and Moore, 2003). The effect on pitch of shifting all harmonics in a harmonic complex tone (H) upwards by a fixed amount in hertz has been determined. For example, starting with an H tone with components at 1000, 1100, 1200, and 1300 Hz (fundamental frequency, *F0* = 100 Hz), a frequency-shifted (inharmonic, I) tone can be created by shifting all components upwards by 25 Hz, giving components at 1025, 1125, 1225, and 1325 Hz. The frequency shift does not change the envelope repetition rate of the sound, and it results in only very small changes in the excitation pattern of the sound (Marmel et al., 2015). Also, the phases of the components are chosen randomly for every stimulus, so the shape of the envelope changes randomly from one stimulus to the next, and does not provide a cue for discriminating the H and I tones. However, the frequency shift does result in a change in the pitch of the sound.

**Fig. 2:**
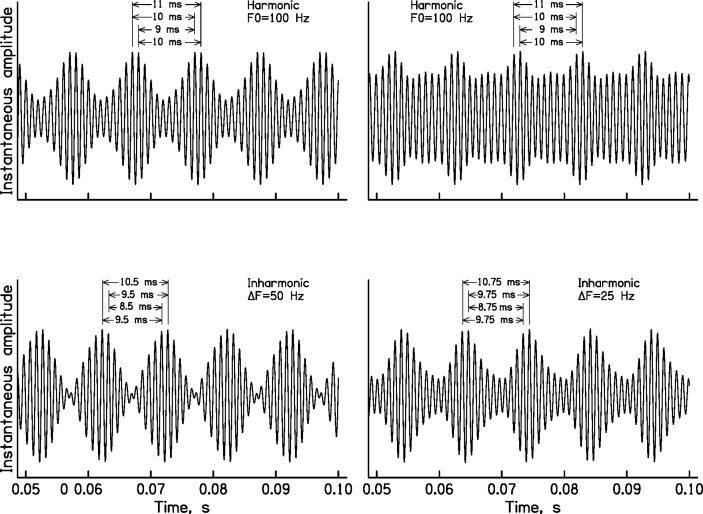
Waveforms of harmonic (H) tones (top) and inharmonic (I) tones (bottom) at the output of a simulated auditory filter centered at 1000 Hz. The H and I tones have the same envelope repetition rate but differ in the time intervals between peaks in the TFS close to adjacent envelope maxima, as indicated by the arrows.

Fig. 2 illustrates how the perceived pitch can be predicted from TFS_n_. The figure shows waveforms of H and I tones at the output of a simulated auditory filter centered at 1000 Hz. The *F0* of the H tones was 100 Hz. The perceived pitch can be predicted based on the following assumptions: (1) most nerve spikes tend to be synchronized to the largest peaks in the TFS on the basilar membrane (TFS_BM_), and these occur close to the envelope peaks, as illustrated by the vertical lines in Fig. 2; (2) the pitch corresponds to the most prominent time intervals between nerve spikes (excluding the very short intervals corresponding to immediately adjacent peaks in TFS_n_); (3) these most prominent intervals correspond to the intervals between peaks in TFS_BM_ close to adjacent envelope peaks on the basilar membrane, as illustrated by the arrows in Fig. 2. For the two H tones (top), the most prominent time interval is 10 ms (1/*F0*). When the harmonics are shifted by 50 Hz (bottom left), the most prominent time interval is 9.5 ms, while when the shift is 25 Hz (bottom right) the most prominent interval is 9.75 ms. In all cases, the perceived pitch corresponds approximately to the reciprocal of the most prominent interval.

Moore and Sek (2009b) used stimuli like those described above to assess the smallest detectable frequency shift at high frequencies, using a test called the TFS1 test (Moore and Sek, 2009a). The value of *F0* was 800 or 1000 Hz and the harmonic number of the lowest harmonic in the passband was 12. It was estimated that the lowest audible component in the stimuli fell at 8000 Hz for *F0* = 800 Hz and 10000 Hz for *F0* = 1000 Hz. For *F0* = 800 Hz most subjects could perform the task consistently. For *F0* = 1000 Hz, about half of the subjects could perform the task consistently. These results suggest that TFS_n_ information can be used by some subjects for pitch discrimination when the frequency of the lowest audible component is 8000 Hz or even 10000 Hz.

A concern with the TFS1 test is that subjects might perform the task using weak excitation-pattern cues rather than TFS_n_ cues. Evidence that excitation pattern cues are not used includes:

1. Excitation patterns broaden with increasing level, reducing resolution of harmonics, but performance of the TFS1 test does not worsen with increasing level, unless the level is very high (Moore and Sek, 2011; Marmel et al., 2015).
2. Randomly varying the level of each component from one stimulus to the next strongly disrupts excitation pattern cues but does not adversely affect performance of the TFS1 test (Jackson and Moore, 2014).
3. Performance predicted using excitation-pattern models is strongly adversely affected by decreasing the signal-to-noise ratio, but human performance is only slightly affected.

I conclude that the most plausible interpretation of the results is that performance of the TFS1 test depends on the use of TFS_n_ cues, and that these cues are available for frequencies up to about 8000 Hz.

It should be noted that there may be marked individual differences in the ability to use TFS information and in the upper frequency limit at which TFS information can be used. Both increasing age and mild hearing loss adversely affect the ability to use TFS cues (Hopkins and Moore, 2007; Füllgrabe et al., 2015). Even among subjects with normal audiograms there is marked individual variability both in the increase in DLF/*f* as *f* is increased from 2000 to 8000 Hz and in the DLF at 8000 Hz (see Fig. 2 in Rose and Moore, 2005). Similarly, performance of the TFS1 test varies across normal-hearing subjects by a factor of 8 or more for *F0*s in the range 50–400 Hz (see Fig. 8 in Moore and Sek, 2009a). For an F0 of 1000 Hz, for which the lowest audible component fell at 10000 Hz, Moore and Sek (2009b) found that four out of eight normal-hearing subjects could reliably complete the adaptive procedure used in the TFS1 test, suggesting that some subjects can use TFS information even at 10000 Hz. The highest frequency at which TFS information can be used binaurally (to detect changes in interaural phase) also varies across young normal-hearing subjects, but only by a factor of about 1.5 (Füllgrabe et al., 2017). The individual differences may reflect both the precision of the neural coding of TFS cues (Marmel et al., 2013), and differences in the ability to make use of those cues.

### 2.2 What key future experiments would help resolve the issue?

Direct recordings from single neurons of the auditory nerve in humans, perhaps made during the course of surgery for an unrelated disorder, could be critical.

### 2.3 What experimental finding would cause you to change your mind?

There have been estimates of the upper limit of phase locking in humans, based on the measurement of mass potentials recorded from a needle that was passed through the eardrum and rested on the cochlear bony capsule (Verschooten et al., 2018). The recorded potentials reflect a mixture of cochlear microphonic (CM) and neural responses. To isolate the neural responses, the recorded response is compared for a tone probe presented alone and for that same tone preceded by an intense masker that is assumed to adapt neural responses but not to adapt the CM. Some “corrections” are applied to compensate for any residual response to the masker, and the difference between the two responses is taken, giving an estimate of the neural component of the response. The estimated neural response is additionally corrected to compensate for the effects of noise in the responses.

These measurements suggest that the upper limit of phase locking in humans is comparable to that measured in cats and is well below 8000 Hz. However, the placement of the recording needle leads to a small signal, with a relatively poor signal-to-noise ratio. Also, the method is based on assumptions and “corrections” that may not be exactly correct. If direct recordings from single neurons of the auditory nerve in humans showed that phase locking ceased to be measurable for frequencies well below 8000 Hz, that would seriously undermine my position.

## 3 Andrew Oxenham

### 3.1 What is the highest frequency for which phase locking is used to code temporal fine structure in humans?

Stimulus-driven spike timing in the auditory nerve, leading to phase-locked responses to acoustic stimulation, is one of the wonders of the nervous system. Because of our auditory system's exquisite temporal sensitivity, differences between the arrival of sound at one ear and its arrival at the other ear can be detected down to tens of microseconds. This astounding feat is made possible not only by the phase-locked responses in the auditory nerve but also by specialized adaptations further up in the system, such as the uniquely large end bulbs of Held that maintain timing fidelity from the auditory nerve into the brainstem. It is reasonably well established that our sensitivity to such phase locking to the TFS of stimuli is limited to frequencies below about 1500 Hz. We know this because our ability to discriminate interaural time differences (ITDs) in pure tones worsens dramatically somewhere between 1000 and 1500 Hz, and is essentially nonexistent beyond 1500 Hz (e.g., Brughera et al., 2013). Whether phase locking in the auditory nerve exists beyond that frequency limit is moot in terms of binaural processing, because it is clear that we cannot use it.

The question addressed here is whether phase-locked responses to TFS can be used for any other kind of processing, possibly involving just monaural input, such as the extraction of pitch. In terms of auditory processing abilities, the most comparable behavioral measurement to pure-tone ITD discrimination thresholds would be the simple frequency difference limen (FDL) for pure tones. Here too, a degradation has been observed at higher frequencies (e.g., Moore, 1973; Wier et al., 1977); however, the degradation with increasing frequency appears much less severe than in the binaural case. In fact, a meta-analysis of FDL studies concluded that the relationship between the log-transformed FDL (in Hz) and frequency (*f*, in kHz) was best described by a power law with an exponent of 0.8 [i.e., log_10_(FDL) = *βf*^0.8^ + *k*] from the lowest commonly measured frequency of 125 Hz up to the highest commonly measured frequency of 8000 Hz (Micheyl et al., 2012). This pattern of results is very different from the rapid deterioration found in the binaural system, which might suggest a different source of limitation.

The fact that FDLs seem to plateau (when expressed as a proportion of frequency) above about 8000 Hz has been cited as indirect evidence that phase locking may be effective up to 8000 Hz, with a coarser place-based (tonotopic) code being used to represent only frequencies above 8000 Hz (Moore and Ernst, 2012). Studies of frequency-modulation (FM) detection and discrimination have used a loss of phase locking to explain differences between performance at low and high carrier frequencies, but in these cases the transition between a timing-based code and a place-based code has been postulated to occur at a frequency below 4000 Hz in order to explain the available data (e.g., Moore and Sek, 1995). It is not clear if these two lines of evidence, pointing to transition frequencies more than an octave apart from each other, can be reconciled within the same computational framework.

Regardless of whether the limit of useful phase locking for frequency coding is postulated to be around 4000 or 8000 Hz, the argument for such a limit has been that psychophysical performance degrades in some way above that frequency limit, thereby reflecting this peripheral coding constraint (Heinz et al., 2001). However, recent work has suggested that FDLs at very high frequencies may not be limited by peripheral coding, and may instead reflect more central constraints, due perhaps to the lack of everyday exposure to very high-frequency pure tones (Lau et al., 2017). Just as a greater proportion of the primary visual cortex is devoted to the foveal than the peripheral visual field, it is possible to speculate that the human auditory cortex may devote more processing capacity (and hence volume) to the lower frequencies (and pitches) that are more commonly encountered in our natural environment.

Another argument for the use of phase locking to code frequency or TFS is that our sensitivity to changes in frequency (including sensitivity to slow rates of FM at low carrier frequencies) seems too good to be explained by the tonotopic (rate-place) code established along the basilar membrane. Taking existing models of cochlear filtering, one can assume that the level fluctuations produced by the FM at the output of at least one filter within the filterbank (i.e., one place on the excitation pattern produced along the basilar membrane) must exceed a certain “threshold” level (e.g., around 1 dB, to be in line with results from amplitude-modulation, AM, detection thresholds). This approach leads to predictions that are similar to those outlined in Zwicker's classic work (Zwicker, 1952, 1956) and that are too high (i.e., too poor) to explain FM detection thresholds, particularly at slow modulation rates and low carrier frequencies. However, these models either assume that detection is based on the output of a single place, or that the information from multiple places is independent. A recent modeling study has shown that if some correlation in firing rate is assumed between cortical neurons tuned to nearby frequencies, then the model's threshold predictions for both FM and AM detection can match human data, even when using the same model parameters for both cases (Micheyl et al., 2013). In other words, by taking advantage of some properties of noise correlation (Cohen and Kohn, 2011), the model results show that it is indeed possible to account for both FM and AM sensitivity using just a rate-place code with no assumed sensitivity to the TFS. This type of model can in principle also explain why slow FM rates produce lower thresholds than high FM rates (Moore and Sek, 1995): any mechanism relying on correlations in firing rates will require a minimum time window over which to evaluate the correlations. As an example, if the minimum time window to evaluate correlations happened to be 100 ms in duration, then correlations would become less important for modulation rates higher than 5 Hz, because the window would extend over more than half a period of the modulation. Beyond 10 Hz, more than a whole modulation period (covering a frequency maximum and minimum) will fall within the window, making it impossible to use this window to reliably detect changes in instantaneous frequency.

Finally, a long-standing impediment to accepting phase locking to TFS as a viable method for processing monaural frequency information is the lack of any physiological candidates to carry out the computation. In contrast to binaural processing, where coincidence and ITD-sensitive cells have been identified and thoroughly studied (Brand et al., 2002; Carr and Konishi, 1990; McAlpine et al., 1995, 2001), there is no indication of a monaural neural mechanism to measure the time intervals between spikes with the precision required to explain human pitch perception. Some studies have hypothesized an across-frequency network of coincidence counters, relying on the phase shifts imposed by cochlear filtering to produce the necessary delays (e.g., Loeb et al., 1983; Shamma and Klein, 2000). Although attractive, this too remains speculative and such a model may not be robust to changes in stimulus level – a criticism that can also be raised against rate-place models.

In summary, the only clear perceptual evidence we have surrounding the effective upper limit of phase locking comes from binaural studies that suggest a limit of less than 1500 Hz. Although higher limits, ranging from 4000 to 8000 Hz have been suggested from perceptual studies of pitch and FM, it remains unclear whether phase locking is even required to account for such data. In fact, essentially every monaural phenomenon postulated to involve phase locking can in principle be accounted for within the framework of a rate-place model that requires no phase locking to TFS. Although there remains some evidence both for and against phase-locking-based theories of frequency coding (Oxenham, 2018), the evidence from perceptual studies is not yet sufficient to inform the effective upper limit of any putative phase-locking mechanism.

### 3.2 What key future experiments would help resolve the issue, and what experimental finding would cause you to change your mind?

It is hard to think of results from any perceptual experiment that would provide strong evidence for the monaural processing of TFS via phase locking. This is because the results from essentially every existing monaural experiment designed to utilize phase-locked temporal information could also be accounted for in principle by a rate-place code that is insensitive to phase locking to the stimulus TFS (e.g., Oxenham et al., 2009). The ability to explain the same data via these alternative mechanisms remains possible because considerable uncertainty remains surrounding the limits of phase locking, the bandwidth and exact shape of the human cochlear filters (Sumner et al., 2018), as well as the mechanisms used to extract and represent both timing and rate-place information at high levels of the auditory system (Micheyl et al., 2013).

Animal experiments could begin to resolve the issue by identifying a potential anatomical and/or physiological substrate for extracting monaural TFS information from the auditory nerve. A viable substrate would likely have to occur at a relatively early stage of processing, such as the cochlear nucleus or possibly inferior colliculus, where the fidelity of timing information remains relatively high. Such a substrate might involve delay lines that, instead of connecting inputs from opposite ears, act to connect inputs from the same ear with delayed versions of themselves, essentially implementing an autocorrelation function. Nothing approximating such a mechanism has yet been reported, despite extensive studies of the auditory brainstem and midbrain. Nevertheless, an indication for the existence of such a mechanism in a non-human species would render the existence of a similar mechanism in humans much more likely, and would therefore provide a much stronger basis for plausibly claiming that TFS is coded via phase locking in the auditory nerve. Such evidence would provide much-needed physiological support for claims that phase locking to TFS is used for monaural processing.

## 4 Michael Heinz

### 4.1 What is the highest frequency for which phase locking is used to code temporal fine structure in humans?

Despite a long history, the debate over whether rate or temporal information is used by humans for the perception of sound remains an active topic of discussion. Because the strength of phase locking diminishes as frequency increases (Johnson, 1980; Palmer and Russell, 1986), dogma in the field has been that phase-locking information exists (and may be used) up to ∼4000 Hz, but above 4000 Hz no temporal information exists and thus rate-place information is used. The approach presented here quantitatively integrates data from animal and human studies via computational modeling to suggest that this dogma is not correct, and to provide evidence suggesting that humans can in fact (and in at least some cases do) use phase-locking information at much higher frequencies than is commonly believed. In this debate, the importance must be noted of distinguishing between the questions of “can humans ever use phase locking at high frequencies?” and “do all humans always use phase locking to code temporal-fine-structure information at high frequencies?”; this section primarily addresses the former question for a simple perceptual task for which animal and human data have been integrated quantitatively.

The classic data on phase locking in auditory-nerve fibers show vector strength (or synchronization index) values that follow a low-pass pattern (Johnson, 1980; Palmer and Russell, 1986), with maximal phase-locking strength at frequencies up to 1000–3000 Hz and a consistent decrease (roll off) as frequency increases above this corner frequency (typically falling into the noise floor around 4000 Hz). These data from cats and guinea pigs are the basis for the dogma that temporal information does not exist above ∼4000 Hz in neural responses. In relating animal data to human performance, at least two questions are critical to consider: 1) how does human phase locking compare (qualitatively and quantitatively) to phase locking in laboratory animals? and 2) is the dogmatic interpretation correct that no temporal information exists once vector strength falls into the noise floor?

The species-difference issue is currently impossible to answer with certainty, because no recordings have been made from human auditory-nerve fibers due to ethical reasons. However, examination of the similarities and differences in phase locking across mammalian species for which data do exist suggests what we can expect to be similar and possibly different in human phase locking. A comparison of phase locking across numerous species by Weiss and Rose (1988a) found that the frequency dependence in all species was well described by a low-pass filter. The important parameters to consider in the low-pass description of phase locking are: 1) maximal strength of phase locking in the low-frequency passband, 2) corner frequency (where phase locking begins to roll off from the maximal value), and 3) slope of the roll off (corresponding to the order of the low-pass filter). It is important to note that the key parameter of discussion in this debate, the “upper limit” of phase locking (the frequency above which no phase-locking information exists), is not a parameter of the low-pass filter description; vector strength appears to simply be reduced as frequency increases consistent with a natural low-pass filter. The data from Weiss and Rose (1988a) suggest that the maximal phase-locking strength and the roll-off slope are consistent across species, with only the low-pass corner-frequency parameter varying across species. This finding is consistent with the fundamental biophysical mechanisms that are believed to contribute to the low-pass nature of temporal coding (e.g., hair-cell membrane resistance/capacitance, synaptic processes) (Kidd and Weiss, 1990; Weiss and Rose, 1988b). Thus, unless humans are truly unique among species used in auditory studies, it is parsimonious to assume that human phase locking will follow a low-pass shape, similar to other species, but with perhaps a different corner frequency. Indirect, non-invasive electrophysiological estimates of temporal coding measured from the middle ear suggest that the frequency dependence of human phase locking is similar (both qualitatively and quantitatively) to that for cats, with the high-frequency extent of phase locking quite similar between human and cats when accounting for neurophonic amplitude differences (Verschooten et al., 2018; see Fig. 5C).

**Fig. 3:**
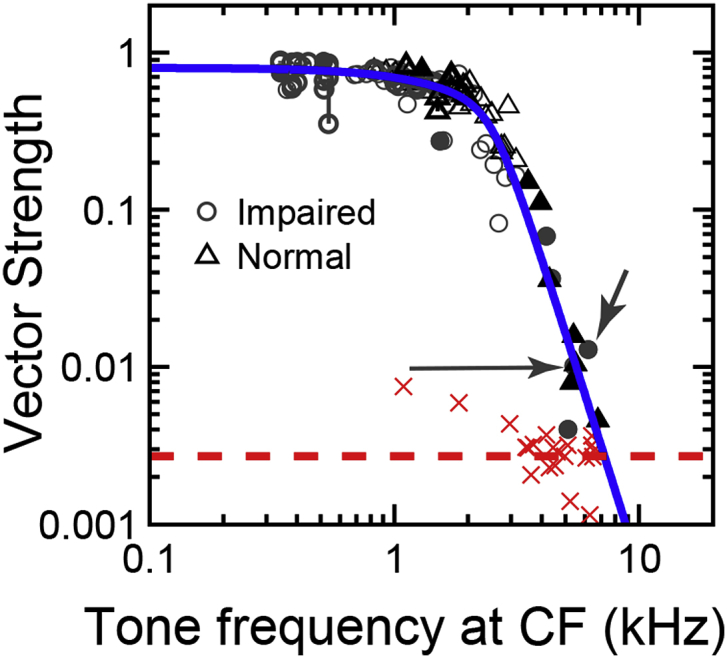
Significant phase locking can be observed up to at least 7000 Hz in chinchilla auditory-nerve fibers when enough data is collected to lower the noise floor for statistical significance. Vector strength is plotted as a function of pure-tone frequency (equal to fiber characteristic frequency, CF) for both normal-hearing animals (triangles) and animals with a moderate noise-induced hearing loss (circles; for details of noise exposure, see Kale and Heinz, 2010). Filled symbols indicate high-CF fibers for which the tone was presented at a single sound level just below saturation so that as many driven spikes as possible could be collected (greater than 100,000 in all cases). Dashed red line indicates center of gravity of fiber noise-floor estimates (red crosses). Solid blue line is a low-pass filter function fitted to normal-hearing data. Only significant values of vector strength are shown (Rayleigh uniformity test, p < 0.001). Note that no difference was observed between phase-locking roll off in normal and impaired ears, despite suggested differences from perceptual studies (Moore, 2008). (Figure from Kale, 2011.)

Despite its prominence in establishing dogma, the “upper limit” parameter is in fact ill-defined in the physiological low-pass phase-locking framework. This is because any attempt to define the upper limit for this continually decreasing function depends entirely on the noise floor (which simply depends on the amount of data collected). This is illustrated in Fig. 3, where significant phase locking in chinchilla auditory-nerve fibers is demonstrated up to 7000 Hz, based on typical auditory-nerve fiber experiments in which far more data were collected than in typical studies (>100,000 spikes per fiber) in order to lower the noise floor (Kale, 2011; Kale and Heinz, 2012). It is critical to note that these data do not indicate that chinchillas have a wider range of phase locking than cats, for which 4000–5000 Hz is typically quoted as an “upper limit” based on the classic data from cat (Johnson, 1980); it is simply that by lowering the noise floor, more of the low-pass filter slope can be observed. The lowest statistically significant value of vector strength in the Johnson data was 0.05–0.1, whereas in these chinchilla data it was 0.005. In fact, chinchillas actually have a lower corner frequency than cats (Sayles and Heinz, 2017), which illustrates the important difference between “corner” frequency and the “upper limit.” The upper-limit parameter must be evaluated in a quantitative Super Disco Breakin perceptual framework to address the question of what is the highest frequency at which humans can use phase-locking information for a specific task, based on the low-pass shape of phase locking.

**Fig. 4:**
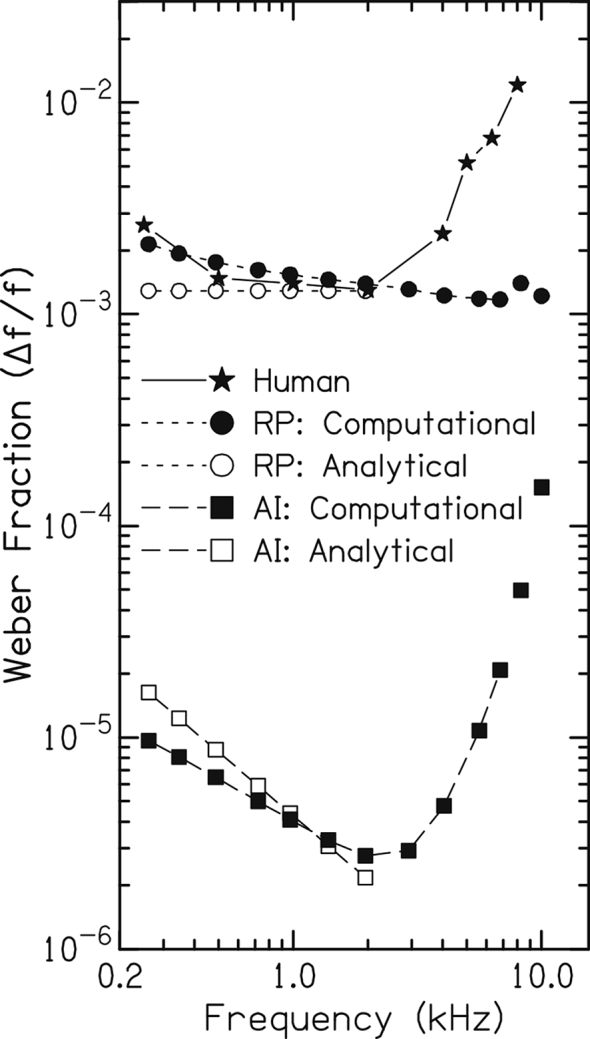
The frequency dependence of human performance on a pure-tone frequency discrimination task matches predicted performance based on rate cues at low frequencies and timing cues at high frequencies (up to 10000 Hz), opposite to common dogma. Predicted performance is based on optimal use of rate-place (RP, circles) and all-information (AI, temporal and rate, squares) cues. The Weber fraction Δ*f*/*f* is plotted as a function of frequency for 200-ms duration tones. Human data from Moore (1973; stars); modeling predictions from an analytical study that did not include the roll off in phase locking (Siebert, 1970; open symbols) and from a computational study that matched the roll off in phase locking to data from cat (Heinz et al., 2001; filled symbols). (Figure from Heinz et al., 2001, with permission.)

Given the established dogma for perceptual tasks in which temporal information is believed to be important, one would expect human performance to follow predictions based on the use of temporal cues up to 4000 Hz, and then to follow rate-place predictions for frequencies >4000 Hz. Analytical predictions of perceptual performance limits on a simple pure-tone frequency discrimination task have been made based on the limits imposed by the stochastic properties of auditory-nerve-fiber responses (Siebert, 1970); however, these did not include the critical property of phase-locking roll off. A computational auditory-nerve-fiber model that captures quantitatively the phase-locking roll off in cats (Zhang et al., 2001) was used in the same stochastic framework to make predictions of performance based on rate-place (RP) information alone and all-information (AI, both temporal and rate-place cues) within the entire AN-fiber population (Heinz et al., 2001). Fig. 4 compares these RP and AI predictions to human frequency-discrimination performance as a function of frequency (Moore, 1973) for 200-ms pure tones. Both analytical and computational RP predictions were essentially flat across frequency, based on scaling invariance of cochlear tuning curves, which largely determines the frequency dependence in RP-cue sensitivity to changes in frequency. It is clear that the frequency-independence of RP predictions is in sharp contrast to human performance above 2000 Hz, which degrades systematically as frequency increases. In contrast, temporal predictions degrade as frequency increases above 2000 Hz similarly to human performance, based on the phase-locking roll off that is observed in all species (Weiss and Rose, 1988a). Based on the similarity in phase-locking maximal strength and roll-off slope across species, this pattern of predictions is not expected to differ between cats and humans, except perhaps for the exact corner frequency where performance would degrade (which does not appear to be very different, if at all, between humans and cats; Verschooten et al., 2018, Fig. 5C). These modeling predictions based on cat phase-locking data suggest that the degradation in frequency-discrimination performance as frequency increases is consistent with the ability of human listeners to use phase-locking information at high frequencies (up to ∼10000 Hz).

It should be noted that while the single-unit AN-fiber data in Fig. 3 have been criticized for requiring an unrealistic number of spikes (∼100,000) collected over many minutes of recording numerous repetitions of the stimulus (see section by Shamma), the predictions in Fig. 4 are based on spikes collected only from a single repetition of a 200-ms tone (but over the entire population of AN fibers). Multiple stimulus repetitions are typically used in neurophysiological studies of single AN fibers, but mimic an ensemble representation of temporal information that exists within the AN-fiber population (i.e., the volley theory; Wever, 1949). For example, for moderate SPLs, many AN fibers saturate at ∼200 spikes/s, which only requires 2500 AN fibers (of 30000 total, i.e., less than 10%) to respond to a single tone repetition in quiet in order to produce a total of ∼100,000 spikes. This rough computation simply demonstrates that this large number of spikes does occur near CF (∼1/10th of the cochlea is about an octave range of CFs) in response to a single tone repetition; however, it must be acknowledged that there is no evidence for any cell types within the cochlear nucleus with as many as ∼2500 inputs. Even if temporal information is not processed as efficiently as these optimal-performance predictions represent, the AI predictions are in fact much better than human performance (as discussed later) and the qualitative trends of these AI and RP predictions would not change significantly.

With all of the parametric dependences on the absolute values of the AI and RP predictions, it was not warranted to estimate an exact “upper limit” for human perception; however, the qualitative trends in Fig. 4 lead to a clear suggestion that, above some frequency, the Weber fractions for frequency discrimination should become flat with frequency when there is insufficient temporal information relative to rate-place cues. This was tested in humans by measuring pure-tone frequency discrimination at even higher frequencies, and the expected pattern was in fact observed, with flat Weber fractions above 8000–10000 Hz (see Fig. 1; Moore and Ernst, 2012).

It must be noted that the predicted AI performance limits based on temporal and rate information were far better than human performance, whereas RP predictions were in the ballpark of human performance (just with the wrong frequency dependence). These are predictions of optimal performance with some uncertainty in the efficiency with which neural mechanisms can extract temporal information and in exact cochlear parameters (e.g., human vs. cat tuning and phase locking), which all could very well affect absolute values but are not expected to affect the qualitative trends. Thus, given the qualitative similarities across species in these parameters, it seems most parsimonious to compare trends between measured and predicted performance. That being said, the exact mechanisms by which temporal information can be decoded in the auditory system remains an important topic for this debate.

In summary, these fundamental predictions of human frequency-discrimination performance as a function of frequency, combined with empirical data from human studies, suggest that humans can make use of phase-locking information up to 8000–10000 Hz on this simple task, and that only rate-place information is relied upon above 8000–10000 Hz. These findings are in fact consistent qualitatively with the common dogma, but differ simply in the “upper limit” of phase-locking usage.

It is also important to qualify that the evidence provided here only suggests that humans can use phase locking at frequencies as high as 8000–10000 Hz in this very simple task. It should not be interpreted to show that *all* humans use phase locking up to 8000–10000 Hz even for this simple task, which requires much training to achieve the performance levels in many published studies (Micheyl et al., 2006). Nor does it suggest that humans can or do use phase locking at these high frequencies in other more complicated tasks. It is merely a feasibility argument that 1) sufficient temporal information exists in AN-fiber responses to support human performance in a simple task at high frequencies, and 2) that the dependence of well-trained human performance on frequency up to 8000–10000 Hz is consistent with the use of phase locking and, importantly, inconsistent with the use of only rate-place information. Given the extensive training required to perform this task well enough to achieve relative frequency-difference limens near 0.15% (Micheyl et al., 2006), it could be that not all humans use phase-locking information up to such high frequencies in all tasks; the simple point here is that they can with sufficient training (e.g., musical training).

### 4.2 What key future experiments would help resolve the issue?

The neurophonic data collected by Verschooten et al. (2018) are a critical step towards evaluating the qualitative and quantitative similarities and differences between humans and cats; however, their data provide an indirect measure of temporal coding in AN fibers. The data that are needed to directly address this critical issue are single-unit recordings from AN fibers in humans; however, significant ethical considerations have prevented these data from being collected to date. The nature of these recordings is highly invasive, with animal studies typically using glass micro-electrodes to record from single AN-fiber axons as the AN bundle exits the brainstem-side of the internal auditory meatus. In humans, several well-established surgical procedures are used to remove tumors on the AN. Theoretically, any procedure that provides access to the cerebello-pontine angle (typically, either by a retro-sigmoid or trans-labyrinthine approach), could provide an opportunity for data collection from individual AN fiber axons prior to tumor removal. Of course, the focus must be on patient health and preservation of hearing, but if ethical considerations could be reconciled, the characterization of phase locking in a few AN fibers in the 2000–5000 Hz CF range would provide invaluable scientific data to inform this ongoing debate. Given the consistent low-pass nature of phase locking in other species, it is likely that very little data would be needed to confirm whether humans are fundamentally similar or different than cats in their phase locking.

### 4.3 What experimental finding would cause you to change your mind?

It is important to note that while the true nature of human phase locking (and tuning) remains uncertain, for the present predictions to change in character (not just quantitatively) would require that the dependence of human phase locking on frequency (and/or the frequency dependence of human cochlear tuning) be fundamentally different than that in cats. For example, uncertainty about the exact phase-locking corner frequency in humans does not change the prediction that temporal-based performance degrades systematically as frequency increases above the corner frequency; to change this fundamental prediction would require human phase locking not to follow a low-pass shape. Likewise, the ongoing debate over whether cochlear tuning is two-to-three times sharper than cats (e.g., Shera et al., 2002; Sumner et al., 2018) does not affect the prediction that rate-place performance does not degrade systematically at frequencies above ∼2000 Hz; rather, a fundamental departure from cochlear scaling invariance would be required to change this prediction. It is possible that recordings from single human AN fibers could demonstrate that the human auditory system is fundamentally different than cats, which would change the present predictions; however, based on recent indirect efforts to compare human and cat tuning and phase locking, these non-parsimonious assumptions do not appear to hold (Verschooten et al., 2018). In fact, the evidence from Verschooten et al. (2018) that human phase locking is in fact not extraordinary, but very much in line with cat data, supports the present argument that phase locking can be used by humans up to 8000–10000 Hz without making any extraordinary assumptions about human temporal coding.

## 5 Shihab Shamma

### 5.1 What is the highest frequency for which phase locking is used to code temporal fine structure in humans?

There is no direct evidence from physiology as to what the maximum phase-locking rates are in human auditory nerve. For the most part, whatever there is seems to show no special high rates beyond 3000–4000 Hz. So, in the absence of that, one is more inclined to believe that the phase locking is limited to this range, because:

1. Animal recordings in the auditory nerve from all common experimental mammals (cats, guinea pigs, ferrets, gerbils) exhibit a limit within this range (Sumner and Palmer, 2012; Evans, 1972; Rose et al., 1967; Johnson, 1980; Taberner and Liberman, 2005; Møller, 1983). Some measurements in owls have suggested higher rates but, even these are weak (Köppl, 1997). Finally, some rodent data, such as those from Heinz's models (Heinz et al., 2001), have suggested higher limits (up to 8000 Hz). Those were backed up by recordings from the Heinz laboratory (see section by Heinz). But these recordings required a large amount of response averaging (thousands of spikes over many minutes of recordings) to lower the noise floor and demonstrate a small phase locking at these high frequencies (see Fig. 3 in section by Heinz). Therefore, such phase-locked responses are functionally impractical, since I am unaware of a natural phenomenon that can average so much data and extract (and hence utilize) such small phase locking. Note also that one in principle can even push up further this high frequency phase-locking limit if one is willing to average over longer durations of tone presentations to reveal even smaller phase-locking amplitudes.

2. Most robust and salient psychoacoustic findings in humans are consistent with a 3000–4000 Hz phase locking limit. For example, the range and salience of the pitch percept, the importance of harmonics that are within the limits of phase locking (Shamma and Klein, 2000; Moore, 2008), the weakness of the pitch induced by high harmonics (3000–4000 Hz) as phase-locking diminishes, *even if spectrally resolved* (Lau et al., 2017), are all strong evidence for the importance of phase locking for this percept, and also for its limit below 4000 Hz. Localization exploiting ITDs also does not seem to make use of any phase-locked cues beyond 1000–2000 Hz (see section by Oxenham).

### 5.2 What key future experiments would help resolve the issue?

As mentioned above, almost all psychoacoustic evidence suggests a lower limit of phase locking, not exceeding 3000–4000 Hz. And since it is clear from all animal experiments that phase locking is limited to a few kHz, then this question must be resolved with human experimentation so to establish whether higher limits exist. Physiologically, the most definitive measurement to resolve this question is one in which human auditory nerve is accessed directly. That can, for example, presumably be done during surgical procedures to implant Modiolus electrodes in CI subjects, or perhaps during surgical removal of tumors. The measurements need not be from single fibers, but instead multi-unit or evoked potentials will do. There are many attempts currently underway to measure and model cochlear potentials so as to determine if there is any evidence for phase locking beyond a few kHz (e.g., Verschooten et al., 2015, section by Joris and Verschooten).

Another line of evidence can be anatomical. Phase locking to very high frequencies requires tight coupling in human inner hair cells so as to reduce the leakage and maintain the phase-locked potentials inside the cells (Manley and Gleich, 1992; Palmer and Russell, 1986). Such specializations that might be indicative of appropriate time constants or low leakage through hair cell membranes have never been described for human hair cells. One may test this possibility further by examining the electrical properties of human hair cells studied directly in a dish, much like studies underway of human cortical neurons acquired from ablations during neuro-surgical procedures for treatment of epilepsy (Mohan et al., 2015).

### 5.3 What experimental finding would cause you to change your mind?

No animal experiments are forthcoming to challenge the current limits of phase locking. So, the only readily feasible experiments (beyond the physiological ones above) are psychoacoustic tasks that implicate high-frequency phase locking. Examples are the experiments of Oxenham and colleagues with high fundamental frequencies which are provocative because they seem to suggest that resolved high-frequency harmonics (>5000 Hz) can contribute to pitch percepts like the phase-locked low harmonics (Lau et al., 2017; also see section by Oxenham). To adopt this view, one either has to consider the possibility that phase locking to these high frequencies is possible, or give up on much evidence that closely links (periodicity) pitch perception to phase locking (as argued above). However, the contribution of the resolved high-frequency harmonics is fairly subtle, and in fact can be interpreted in terms of spectral pattern recognition processes that do not need any phase-locked information, something akin to the harmonic patterns of the Bat echolocation calls that exceed 60000 Hz (Suga et al., 1978) and hence are clearly not phase locked. So, in short, I cannot readily conceive of a plausible test to falsify the upper limit of phase locking at 3000–4000 Hz!

## 6 Philip Joris and Eric Verschooten

### 6.1 What is the highest frequency for which phase locking is used to code temporal fine structure in humans?

Several kinds of TFS upper frequency limit (UL-FS) can be distinguished: behavioral vs. physiological, monaural vs. binaural, peripheral vs. central. Parsimony suggests that the limit at which the human central nervous system (CNS) can access (“use”) phase locking is ∼1400 Hz, lower than in experimental animal models with low-frequency hearing. We base this assertion on two main arguments, built on experimental observations in animals and humans.

Our first argument is based on binaural physiology and behavior. In short, the argument runs as follows, starting with five observations (see Joris and Verschooten, 2013 for a fuller treatment and references to the primary literature):

1. In diverse binaural tasks, humans show an abrupt UL-FS for binaural sensitivity near 1400 Hz (e.g. Brughera et al., 2013). Cats have a similar binaural “brick-wall” limit, but more than 1000 Hz higher, at about 2800 Hz (Jackson et al., 1996; Wakeford and Robinson, 1974; Heffner HE and Heffner RS, personal communication 2002).
2. In cat, this behavioral binaural limit is very close to the physiological limit, i.e. the highest frequency at which binaural sensitivity to fine structure is observed (2800 Hz) (Joris, 2003; Kuwada and Yin, 1983; Rose et al., 1966) (Fig. 5B). Note that this is several kHz lower than the UL-FS in the auditory nerve (∼5000–6000 Hz; Johnson, 1980) (Fig. 5A).

**Fig. 5:**
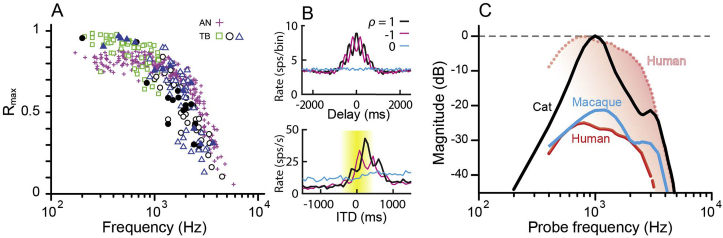
A. Maximal vector strengths of phase locking to CF-tones in auditory nerve (Johnson, 1980) and in bushy cells of the cochlear nucleus, recorded in the trapezoid body (TB). Each symbol indicates one neuron. The TB data are from bushy-type neurons: for meaning of different symbols see Joris et al. (1994a). Note the higher values in TB than in AN at low frequency, and the reverse at high frequency. B. Autocorrelograms for an auditory nerve fiber with characteristic frequency (CF) of 3200 Hz (top) and interaural time delay (ITD) functions for an IC neuron (CF = 2790 Hz) (bottom), in each case to correlated (ρ = 1), anti-correlated (ρ = −1) and uncorrelated (ρ = 0) broadband noise. The small oscillations reflect phase locking to fine structure, and the dominant broad mound reflects phase-locking to envelope generated by cochlear filtering. The yellow shading indicates roughly the range of acoustic delays for cat: there are several peaks of the fine-structure oscillation within this range, indicating spatial aliasing. (Modified from Joris, 2003.) C. Trendlines for phase locking to fine structure measured via the neurophonic recorded in the middle ear of cat, macaque monkey, and normal-hearing humans. The solid lines show magnitude re maximum in cat; the dotted line shows the human trendline normalized to maximum in cat. (Modified from Verschooten et al., 2018.)
3. Binaural pathways are extremely specialized to make use of fine structure. This is not only the case for primary binaural neurons but also for monaural neurons which supply them with phase-locked inputs. Striking specializations, likely costly in energy demands (Attwell and Laughlin, 2001), occur in the size and placement of synaptic terminals, in membrane properties, and in patterns of innervation (Young and Oertel, 2004). They are revealed in binaural but also monaural neural responses to stimulus fine structure, e.g. in the strength and frequency-extent of phase locking (Joris et al., 1994a) (Fig. 5A).
4. Of the monaural brainstem pathways that project elsewhere (referred to here as “non-binaural pathways”), none match the UL-FS observed in the binaural pathway. Of all projection neurons of the cochlear nucleus, the UL-FS is highest in bushy cells, which project to the primary nuclei of binaural interaction. Even that limit is somewhat lower than in the auditory nerve (Fig. 5A) (references in Joris and Verschooten, 2013).
5. Finally, the human auditory brainstem shows basic cytoarchitectural and cytological similarities to that of laboratory animals. Admittedly, human tissue lacks the quality and possibilities for fine-grained comparative studies, but at a superficial level, the human brainstem does not display marked differences in neuroanatomy suggestive of unusual temporal specializations. Note that such features are observed in cetacean brains, where the quality of material is also nonoptimal (Zook and DiCaprio, 1990).

Putting the above observations together, the prediction is that the UL-FS in the human auditory nerve is lower than in the cat. The reasoning boils down to the following: the binaural system, which is the only system for which we can be absolutely sure that it makes use of TFS, squeezes out monaural temporal coding to the fullest towards creation of binaural temporal sensitivity, so if that system cannot make use of fine-structure information above a certain limit, then no other system can. In cat, the most relevant limits are roughly 2800 Hz (upper binaural behavioral and physiological limit) and 5000–6000 Hz (auditory nerve). This downshift of more than 2000 Hz is due to synaptic convergence and integrative membrane properties: about 1000 Hz is “lost” between bushy cell output and its auditory nerve input, and another 1000 Hz in ITD sensitivity (presumably between bushy cells and primary binaural neurons). In human the behavioral limit is only 1400 Hz (compared to 2800 Hz in cat, almost 1500 Hz higher), so that we predict a limit in the auditory nerve about 1500 Hz lower than in cat, i.e. about 3500–4500 Hz. One may squabble regarding the experimental values, which are not as hard as implied by a single number, but the exact numbers are not critical to our argument; rather, it is the direction of the trends which matters here. The data in cat are the most complete, but data in other species (gerbil, guinea pig, chinchilla, rabbit, macaque) are qualitatively in line with these.

The suggestion that humans have access to fine structure coding up to frequencies as high as 8000–12000 Hz for monaural perception (Alves-Pinto and Lopez-Poveda, 2005; Heinz et al., 2001; Moore, 2014; Moore and Sek, 2009b), while binaural sensitivity to fine structure drops out at 1400 Hz, is difficult to square with the available animal physiology and anatomy. We assert that the low binaural UL-FS in humans reflects a peripheral limitation, i.e. in the auditory nerve. The alternative is that the binaural UL-FS reflects a central limitation specific to the binaural pathway. Recordings from the human auditory nerve in neurosurgical patients (Møller and Jho, 1989) seemed to be a way to examine this issue. While doing the groundwork for such recordings (Verschooten and Joris, 2014), we found – much to our surprise but already described by others (Henry, 1995) – that the “cochlear microphonic” recorded in experimental animals, e.g. at the round window, contains a sizeable contribution from phase-locked neural generators. This provides the opportunity to study human phase locking directly in healthy, normal-hearing volunteers using a trans-tympanic recording approach to the cochlea, initially developed in patients (Eggermont, 1977; Harrison et al., 1981). We first studied the cochlear potential to sustained stimuli in animals, and developed a stimulus and analysis paradigm that allowed us to tease out the neural contribution. Disambiguation of this component from the signal generated by hair cells relies on neural adaptation. Verschooten et al. (2015, 2018) found that the UL-FS of the neural contribution to the AC signal recorded near the cochlea (“neurophonic”), was quite consistent with (somewhat lower than) single-fiber data, both in cat (4700 Hz) and macaque (4100 Hz); the limit measured in humans was still lower: 3300 Hz (Verschooten et al., 2015, 2018) (Fig. 5C). A general issue with statements on upper limits is the measurement noise floor: because the amplitude of the neurophonic is much smaller in humans and macaque than in cats, it is possible that a lower limit reflects a poor signal-to-noise ratio. One counterargument is that the nerve limit in macaque estimated by the neurophonic is quite close to that obtained from single-fiber nerve recordings, and arguably the monkey data are most relevant for conclusions re humans. Even a conservative (and in our view less plausible) interpretation of these data, which normalizes maxima across species, shows that at best human phase-locking does not have a higher upper-limit than cat or monkey (Fig. 5C).

An often-stated counterargument against using the binaural UL-FS as a general index for use of fine structure, is that it is determined by the acoustic head width. Mammals sample sounds with two independent and spatially separate receptors. Their distance sets a spatial-aliasing limit: at sound wavelengths smaller than twice the acoustic head width, ITD become ambiguous. Thus, the argument goes, the binaural use of fine-structure evolved (phylogenetically and/or ontogenetically) to a frequency limit where it ceases to be useful for sound localization, and this limit depends on the species, being higher in small-headed animals. There is no space here to fully address the merit of this reasoning and its experimental basis (Hartmann and Macaulay, 2014; Heffner et al., 2001), but we have the following remarks. First, temporal differences are not only useful for azimuthal sound localization: they also serve binaural detection. Even when the ITD does not point to a unique azimuth, it still informs the brain about binaural differences. Second (and perhaps related to the first point): the frequency of spatial aliasing is actually much lower (by an octave!) than the binaural UL-FS. It corresponds to ∼700 Hz in human (Hartmann and Macaulay, 2014) and 1400 Hz in cat. Third, the ambiguity only holds for narrowband signals (Trahiotis and Stern, 1994). Note that azimuthal sound localization in the barn owl is dominated by frequencies of 5000–9000 Hz (Konishi, 1973): this range is entirely above its spatial aliasing limit (∼4000 Hz, based on an acoustic head width of ±250 μs), yet barn owls seem to manage well. Fourth, the head width counterargument does not negate the physiological and anatomical observations made in animals. Even if the upper limit of binaural sensitivity is determined by head width, this does not invalidate the lower UL-FS in non-binaural than in binaural pathways, observed in animals.

In the context of phase locking in humans, the head width argument implicitly suggests a dissociation and even reversal of the binaural and monaural UL-FS: a scenario where the binaural UL-FS decreases with increasing head size but where the UL-FS in non-binaural pathways evolves independently and can increase to frequencies as high as 8000–12000 Hz. However, our neurophonic recordings suggest that with increasing head size, *both* the binaural and peripheral UL-FS limits decrease. The macaque has a larger head width than cat, and its behavioral ITD discrimination shows an upturn in threshold at about 2000 Hz (Scott et al., 2007), which is indeed lower than in cat and higher than in human. However, the upper frequency limit of single-fiber phase locking in the auditory nerve of macaque is also shifted downward compared to cat (Verschooten et al., 2018).

When ITD sensitivity is studied in response to broadband noise, fine structure loses its potency relative to envelope at a lower frequency than suggested by the binaural UL-FS to pure tones (Devore and Delgutte, 2010; Joris, 2003). It was remarked that the transition of dominance of fine structure to envelope occurs at a CF range near the frequency of spatial aliasing (Joris, 2003). A similar transition was reported for humans, but at frequencies about an octave lower (Bernstein and Trahiotis, 1996). Perhaps the low binaural UL-FS in large (-headed) animals is related to their low vocal fundamental frequencies with harmonics that become unresolved at lower CFs than is the case for vocalizations of small animals.

In summary, while there is some experimental evidence for a lower UL-FS in the binaural system of animals with larger head width, the causal nature of that association is unclear. Moreover, we are not aware of any physiological evidence, in any species, showing a higher UL-FS in non-binaural than in binaural pathways.

There have been occasional physiological reports of nerve phase locking to high frequencies. Rose et al. (1967) mention “isolated instances” of phase locking as high as 12000 Hz in squirrel monkey. Teich et al. (1993) found phase locking to pure tones as high as 18000 Hz in cat. Recio-Spinoso et al. (2005) found phase-locking up to 12000 Hz in first-order Wiener kernel analysis of responses to broadband noise in chinchilla, and pure-tone phase locking up to 10000 Hz (Temchin et al., 2005). Phase locking up to 7000 Hz in chinchilla was also reported by Kale and Heinz (2012) (shown in Fig. 3). Over the years, we have used a variety of stimuli and recording and analysis methods but have not observed convincing cases of such phase locking. We are therefore skeptical of its existence and plan further experiments to examine this issue. Even if it exists, it can be questioned what phase locking means if it is only revealed after exhaustive averaging or analyses that are not available to the CNS. If the claim is that CNS neurons can extract weak phase locking from pooled inputs, this should be evident in CNS recordings but has not been observed: UL-FS in the CNS are invariably lower than in the auditory nerve. We would rather emphasize that population characterizations of phase-locking (Blackburn and Sachs, 1989; Johnson, 1980; Joris et al., 1994a; Rhode and Smith, 1986; Winter and Palmer, 1990) are optimistic. The values reported are typically maximal vector strengths to the sustained response portion to pure tones, measured over a range of SPLs and for frequencies near CF, and with full knowledge of the stimulus in calculating the vector strength. Values tend to decrease with SPL and the UL-FS is lower for fibers stimulated in their low-frequency “tail” (Joris et al., 1994b). Spurious phase locking is also easily induced by electrical crosstalk, e.g. from earphones (Johnson, 1974).

One possibility allowing a higher monaural than binaural UL-FS, which would not contradict the observations stated above, is that temporal information of the auditory nerve is already transformed into a different, non-phase-locked code in the cochlear nucleus, which could underlie aspects of monaural perception and would not be detectable by traditional measures of phase locking. As an analogy: phase locking to fine structure in auditory cortex is limited to only a few hundred Hz at best, but cortical neurons can show ITD sensitivity at frequencies as high as 2500 Hz (Reale and Brugge, 1990) because they inherit ITD sensitivity from the brainstem, where the temporal information is transformed into a rate code. Similarly, one could envisage a time-to-rate (or other) conversion at the level of the cochlear nucleus so that TFS coding is used to generate a new neural property underlying some aspect of perception, without there being “simple” phase-locking at those frequencies. There is no evidence, that we are aware of, for such a process at frequencies higher than the binaural UL-FS. But absence of evidence is of course not evidence of absence.

### 6.2 What key future experiments would help resolve the issue?

If the debate on place versus temporal codes for pitch is a guide, it seems unlikely that a firm determination of a monaural UL-FS can be settled through behavioral experiments. Direct evidence pinning down the physiological UL-FS would require recordings from the human CNS, but then relating such recordings to perception is not a trivial manner.

In the short term, physiological experiments testing psychophysical interpretations would help, e.g. they could check whether other physiological cues than coding of fine structure can be a basis for perception. For example, the discrimination of harmonic and inharmonic tones has been used as a test for the use of TFS (Moore and Sek, 2009a). Recordings in animals to such stimuli, using stimulus frequencies in a range where no coding of fine structure is observed, can examine whether other response attributes can be identified that are a possible basis for discrimination.

### 6.3 What experimental finding would cause you to change your mind?

The auditory nerve is the bottleneck to the CNS. Phase locking of auditory nerve fibers by itself does not qualify as “neural temporal processing” but is a precondition for such processing in the CNS. Single-fiber data from the human auditory nerve – preferably in normal-hearing subjects – could address whether and how the phase-locking limit in humans differs from that in experimental animals. Convincing evidence of a higher UL-FS in the human auditory nerve than in experimental animals would not constitute proof that phase locking at frequencies higher than in cat is actually used in human perception, but it would leave open the possibility. If the limit is lower than in cat, it leaves no room for such a role. However, we do not see a possibility with current techniques to obtain such single-fiber data.

Perhaps more feasible is to measure an electrophysiological binaural UL-FS in humans. If this were higher than the behavioral UL-FS (1400 Hz), it would indicate that the latter limit is not imposed by the primary site of binaural interaction (as in the cat), but at some higher level of “readout,” leaving open the possibility that fine structure for frequencies above 1400 Hz is available and used in non-binaural pathways.

## 7 Concluding remarks

The contributors have taken a number of different approaches to the questions, from a focus on the underlying neural representations to a focus on perceptual performance, and including comparative results from animal and human studies, and computational modeling. As described by several contributors, phase locking can be used for binaural processing in humans up to about 1500 Hz. In the binaural case we can say with some assurance that phase-locking information is used up to this frequency because no other cues are available. However, there is clearly some disagreement about the general upper limit, which includes monaural TFS processing, with values of ∼1500 Hz (Oxenham, Joris and Verschooten), 3000–4000 Hz (Shamma), and 8000–10000 Hz (Moore, Heinz) proposed by the contributors. The questions posed by Joris and Verschooten are why should, and how can, the limit for monaural TFS coding be greater than that for binaural TFS coding? Proponents of a high upper limit need neurophysiological evidence that these high frequencies are represented by phase locking in humans, *and* that this temporal information is processed in the (preferably human) auditory brain.

The range of frequencies under debate, from 1500 to 10000 Hz, is highly significant for human hearing. This range includes the range of maximum threshold sensitivity, and is of vital importance for speech perception. If phase locking is not used to code frequencies above 1500 Hz, then we are left with some difficult questions. In particular, why is pure tone frequency discrimination, measured in terms of a relative difference limen, so good for frequencies up to 4000–5000 Hz? Indeed, as described by Moore and by Heinz, the relative difference limen continues to increase up to about 8000 Hz. If phase locking is not used up to 8000 Hz, how can place information give such good performance and why can the auditory system not get the same benefit from place information at frequencies above 8000 Hz? Proponents of a low upper limit need evidence that place coding can account for the high fidelity of frequency coding up to 4000–5000 Hz, for the drop off in fidelity at higher frequencies, and for performance on tasks such as the TFS1 test for high harmonic frequencies. As Oxenham suggests, it is possible that there are central constraints that limit performance at high frequencies, perhaps because of the lack of everyday exposure to these stimuli. If this is the case, and the deterioration in discrimination performance does not reflect a change in mechanism, should we consider the possibility that frequency discrimination, and perhaps pitch perception in general, is dependent on place coding at all frequencies?

The contributors suggest a number of experiments that may help to resolve the question of the upper frequency limit. Moore, Heinz, Shamma, and Joris and Verschooten, all suggest that recordings from the human auditory nerve would help to determine the maximum frequency for peripheral phase locking. Joris and Verschooten provide recent evidence from human neurophonic recordings that suggests an upper limit in the auditory nerve below 5000 Hz, consistent with that of other mammals. However, Moore questions the assumptions behind this derivation. Direct recordings from the human auditory nerve (preferably single fiber) may be the only way to settle this issue conclusively. Furthermore, Heinz argues that the neurophonic data of Verschooten et al. (2018) are in fact consistent with a high upper limit, of perhaps 10000 Hz. The argument rests on whether phase locking at these high rates can be extracted from the noise floor, as suggested by Heinz and disputed by Shamma and by Joris and Verschooten. Shamma suggests that information regarding the upper limit may also be obtained by studying the electrical properties of human inner hair cells *in vitro.*

Even if the peripheral frequency limit can be determined, the existence of a temporal representation in the auditory nerve does not imply that the information is used by higher centers. Animal experiments that demonstrate the use of monaural TFS information (Oxenham) would help to confirm that such information can be utilized in humans, and perhaps suggest an upper frequency limit. Alternatively, electrophysiological binaural recordings in humans may provide evidence of a higher upper limit than expected based on behavioral studies (Joris and Verschooten). Such studies may not give a definitive answer, but they will provide further fuel to a debate that is enriched (or perhaps cursed) by strong arguments from all viewpoints.

## References

[bib1] Alves-Pinto A., Lopez-Poveda E.A. (2005). Detection of high-frequency spectral notches as a function of level. J. Acoust. Soc. Am..

[bib2] Attneave F., Olson R.K. (1971). Pitch as a medium: a new approach to psychophysical scaling. Am. J. Psychol..

[bib3] Attwell D., Laughlin S.B. (2001). An energy budget for signaling in the grey matter of the brain. J. Cereb. Blood Flow Metab. Off. J. Int. Soc. Cereb. Blood Flow Metab..

[bib4] Bernstein L.R., Trahiotis C. (1996). The normalized correlation: accounting for binaural detection across center frequency. J. Acoust. Soc. Am..

[bib5] Blackburn C.C., Sachs M.B. (1989). Classification of unit types in the anteroventral cochlear nucleus: PST histograms and regularity analysis. J. Neurophysiol..

[bib6] Brand A., Behrend O., Marquardt T., McAlpine D., Grothe B. (2002). Precise inhibition is essential for microsecond interaural time difference coding. Nature.

[bib7] Brughera A., Dunai L., Hartmann W.M. (2013). Human interaural time difference thresholds for sine tones: the high-frequency limit. J. Acoust. Soc. Am..

[bib8] Carlyon R.P., Moore B.C.J. (1984). Intensity discrimination: a severe departure from Weber's Law. J. Acoust. Soc. Am..

[bib9] Carr C.E., Konishi M. (1990). A circuit for detection of interaural time differences in the brain stem of the barn owl. J. Neurosci..

[bib10] Cedolin L., Delgutte B. (2005). Pitch of complex tones: rate-place and interspike interval representations in the auditory nerve. J. Neurophysiol..

[bib11] Cohen M.R., Kohn A. (2011). Measuring and interpreting neuronal correlations. Nat. Neurosci..

[bib12] Devore S., Delgutte B. (2010). Effects of reverberation on the directional sensitivity of auditory neurons across the tonotopic axis: influences of interaural time and level differences. J. Neurosci..

[bib13] Eggermont J.J. (1977). Compound action potential tuning curves in normal and pathological human ears. J. Acoust. Soc. Am..

[bib14] Evans E.F. (1972). The frequency response and other properties of single fibres in the Guinea-pig cochlear nerve. J. Physiol..

[bib15] Florentine M. (1983). Intensity discrimination as a function of level and frequency and its relation to high-frequency hearing. J. Acoust. Soc. Am..

[bib16] Füllgrabe C., Moore B.C.J., Stone M.A. (2015). Age-group differences in speech identification despite matched audiometrically normal hearing: contributions from auditory temporal processing and cognition. Front. Aging Neurosci..

[bib17] Füllgrabe C., Harland A.J., Sek A.P., Moore B.C.J. (2017). Development of a method for determining binaural sensitivity to temporal fine structure. Int. J. Audiol..

[bib18] Glasberg B.R., Moore B.C.J. (1990). Derivation of auditory filter shapes from notched-noise data. Hear. Res..

[bib19] Harrison R.V., Aran J.M., Erre J.P. (1981). AP tuning curves from normal and pathological human and Guinea pig cochleas. J. Acoust. Soc. Am..

[bib20] Hartmann W.M., Macaulay E.J. (2014). Anatomical limits on interaural time differences: an ecological perspective. Front. Neurosci..

[bib21] Heffner R.S., Koay G., Heffner H.E. (2001). Sound localization in a new-world frugivorous bat, Artibeus jamaicensis: acuity, use of binaural cues, and relationship to vision. J. Acoust. Soc. Am..

[bib22] Heinz M.G., Colburn H.S., Carney L.H. (2001). Evaluating auditory performance limits: I. one-parameter discrimination using a computational model for the auditory nerve. Neural Comput..

[bib23] Henry K.R. (1995). Auditory nerve neurophonic recorded from the round window of the Mongolian gerbil. Hear. Res..

[bib24] Hopkins K., Moore B.C.J. (2007). Moderate cochlear hearing loss leads to a reduced ability to use temporal fine structure information. J. Acoust. Soc. Am..

[bib25] Jackson H.M., Moore B.C.J. (2014). The role of excitation-pattern and temporal-fine-structure cues in the discrimination of harmonic and frequency-shifted complex tones. J. Acoust. Soc. Am..

[bib26] Jackson L.L., Heffner H.E., Heffner R.S. (1996). Species differences in the upper limit of binaural phase discrimination. Assoc. Res. Otolaryngol. Abs..

[bib27] Johnson D.H. (1974). The Response of Single Auditory-Nerve Fibers in the Cat to Single Tones: Synchrony and Average Discharge Rate.

[bib28] Johnson D.H. (1980). The relationship between spike rate and synchrony in responses of auditory-nerve fibers to single tones. J. Acoust. Soc. Am..

[bib29] Joris P.X. (2003). Interaural time sensitivity dominated by cochlea-induced envelope patterns. J. Neurosci..

[bib30] Joris P.X., Carney L.H., Smith P.H., Yin T.C. (1994). Enhancement of neural synchronization in the anteroventral cochlear nucleus. I. Responses to tones at the characteristic frequency. J. Neurophysiol..

[bib31] Joris P.X., Smith P.H., Yin T.C. (1994). Enhancement of neural synchronization in the anteroventral cochlear nucleus. II. Responses in the tuning curve tail. J. Neurophysiol..

[bib32] Joris P.X., Verschooten E. (2013). On the limit of neural phase locking to fine structure in humans. Adv. Exp. Med. Biol..

[bib33] Kale S. (2011). Temporal Coding in Auditory-Nerve Fibers Following Noise-Induced Hearing Loss.

[bib34] Kale S., Heinz M.G. (2010). Envelope coding in auditory nerve fibers following noise-induced hearing loss. J. Assoc. Res. Otolaryngol..

[bib35] Kale S., Heinz M.G. (2012). Temporal fine structure coding at high frequencies following noise-induced hearing loss. Assoc. Res. Otolaryngol. Abs..

[bib36] Kidd R.C., Weiss T.F. (1990). Mechanisms that degrade timing information in the cochlea. Hear. Res..

[bib37] Konishi M. (1973). How the owl tracks its prey: experiments with trained barn owls reveal how their acute sense of hearing enables them to catch prey in the dark. Am. Sci..

[bib38] Köppl C. (1997). Phase locking to high frequencies in the auditory nerve and cochlear nucleus magnocellularis of the barn owl, Tyto alba. J. Neurosci..

[bib39] Kuwada S., Yin T.C.T. (1983). Binaural interaction in low-frequency neurons in inferior colliculus of the cat. I. Effects of long interaural delays, intensity, and repetition rate on interaural delay function. J. Neurophysiol..

[bib40] Lau B.K., Mehta A.H., Oxenham A.J. (2017). Superoptimal perceptual integration suggests a place-based representation of pitch at high frequencies. J. Neurosci..

[bib41] Loeb G.E., White M.W., Merzenich M.M. (1983). Spatial cross correlation: a proposed mechanism for acoustic pitch perception. Biol. Cybern..

[bib42] Manley G.A., Gleich O. (1992). Evolution and specialization of function in the avian auditory periphery. The Evolutionary Biology of Hearing.

[bib43] Marmel F., Linley D., Carlyon R.P., Gockel H.E., Hopkins K., Plack C.J. (2013). Subcortical neural synchrony and absolute thresholds predict frequency discrimination independently. J. Assoc. Res. Otolaryngol..

[bib44] Marmel F., Plack C.J., Hopkins K., Carlyon R.P., Gockel H.E., Moore B.C.J. (2015). The role of excitation-pattern cues in the detection of frequency shifts in bandpass-filtered complex tones. J. Acoust. Soc. Am..

[bib45] McAlpine D., Jiang D., Palmer A.R. (1995). Interaural delay sensitivity and the classification of low best-frequency binaural responses in the inferior colliculus of the Guinea pig. Hear. Res..

[bib46] McAlpine D., Jiang D., Palmer A.R. (2001). A neural code for low frequency sound localization in mammals. Nat. Neurosci..

[bib47] Micheyl C., Delhommeau K., Perrot X., Oxenham A.J. (2006). Influence of musical and psychoacoustical training on pitch discrimination. Hear. Res..

[bib48] Micheyl C., Xiao L., Oxenham A.J. (2012). Characterizing the dependence of pure-tone frequency difference limens on frequency, duration, and level. Hear. Res..

[bib49] Micheyl C., Schrater P.R., Oxenham A.J. (2013). Auditory frequency and intensity discrimination explained using a cortical population rate code. PLoS Comput. Biol..

[bib50] Mohan H., Verhoog M.B., Doreswamy K.K., Eyal G., Aardse R., Lodder B.N., Goriounova N.A., Asamoah B., Brakspear B., A.C., Groot C., van der Sluis S. (2015). Dendritic and axonal architecture of individual pyramidal neurons across layers of adult human neocortex. Cerebr. Cortex.

[bib51] Møller A.R. (1983). Frequency selectivity of phase-locking of complex sounds in the auditory nerve of the rat. Hear. Res..

[bib52] Møller A.R., Jho H.D. (1989). Response from the exposed intracranial human auditory nerve to low-frequency tones: basic characteristics. Hear. Res..

[bib53] Moore B.C.J. (1973). Frequency difference limens for short-duration tones. J. Acoust. Soc. Am..

[bib54] Moore B.C.J. (2008). The role of temporal fine structure processing in pitch perception, masking, and speech perception for normal-hearing and hearing-impaired people. J. Assoc. Res. Otolaryngol..

[bib55] Moore B.C.J. (2014). Auditory Processing of Temporal Fine Structure: Effects of Age and Hearing Loss.

[bib56] Moore B.C.J., Ernst S.M. (2012). Frequency difference limens at high frequencies: evidence for a transition from a temporal to a place code. J. Acoust. Soc. Am..

[bib57] Moore B.C.J., Gockel H. (2011). Resolvability of components in complex tones and implications for theories of pitch perception. Hear. Res..

[bib58] Moore B.C.J., Moore G.A. (2003). Discrimination of the fundamental frequency of complex tones with fixed and shifting spectral envelopes by normally hearing and hearing-impaired subjects. Hear. Res..

[bib59] Moore B.C.J., Sek A. (1995). Effects of carrier frequency, modulation rate, and modulation waveform on the detection of modulation and the discrimination of modulation type (amplitude modulation versus frequency modulation). J. Acoust. Soc. Am..

[bib60] Moore B.C.J., Sek A. (2009). Development of a fast method for determining sensitivity to temporal fine structure. Int. J. Audiol..

[bib61] Moore B.C.J., Sek A. (2009). Sensitivity of the human auditory system to temporal fine structure at high frequencies. J. Acoust. Soc. Am..

[bib62] Moore B.C.J., Sek A. (2011). Effect of level on the discrimination of harmonic and frequency-shifted complex tones at high frequencies. J. Acoust. Soc. Am..

[bib63] Oxenham A.J. (2018). How we hear: the perception and neural coding of sound. Annu. Rev. Psychol..

[bib64] Oxenham A.J., Micheyl C., Keebler M.V. (2009). Can temporal fine structure represent the fundamental frequency of unresolved harmonics?. J. Acoust. Soc. Am..

[bib65] Oxenham A.J., Micheyl C., Keebler M.V., Loper A., Santurette S. (2011). Pitch perception beyond the traditional existence region of pitch. Proc. Natl. Acad. Sci. Unit. States Am..

[bib66] Oxenham A.J., Shera C.A. (2003). Estimates of human cochlear tuning at low levels using forward and simultaneous masking. J. Assoc. Res. Otolaryngol..

[bib67] Palmer A.R., Russell I.J. (1986). Phase-locking in the cochlear nerve of the Guinea-pig and its relation to the receptor potential of inner hair-cells. Hear. Res..

[bib68] Reale R.A., Brugge J.F. (1990). Auditory cortical neurons are sensitive to static and continuously changing interaural phase cues. J. Neurophysiol..

[bib69] Recio-Spinoso A., Temchin A.N., van Dijk P., Fan Y.-H., Ruggero M.A. (2005). Wiener-kernel analysis of responses to noise of chinchilla auditory-nerve fibers. J. Neurophysiol..

[bib70] Rhode W.S., Smith P.H. (1986). Encoding timing and intensity in the ventral cochlear nucleus of the cat. J. Neurophysiol..

[bib71] Rose J.E., Brugge J.F., Anderson D.J., Hind J.E. (1967). Phase-locked response to low-frequency tones in single auditory nerve fibers of the squirrel monkey. J. Neurophysiol..

[bib72] Rose J.E., Gross N.B., Geisler C.D., Hind J.E. (1966). Some neural mechanisms in the inferior colliculus of the cat which may be relevant to localization of a sound source. J. Neurophysiol..

[bib73] Rose M.M., Moore B.C.J. (2005). The relationship between stream segregation and frequency discrimination in normally hearing and hearing-impaired subjects. Hear. Res..

[bib74] Sayles M., Heinz M.G., Manley G.A., Gummer A.W., Popper A.N., Fay R.R. (2017). Afferent coding and efferent control in the normal and impaired cochlea. Understanding the Cochlea.

[bib75] Scott B.H., Malone B.J., Semple M.N. (2007). Effect of behavioral context on representation of a spatial cue in core auditory cortex of awake macaques. J. Neurosci..

[bib76] Semal C., Demany L. (1990). The upper limit of "musical" pitch. Music Percepn.

[bib77] Semal C., Demany L. (2006). Individual differences in the sensitivity to pitch direction. J. Acoust. Soc. Am..

[bib78] Shamma S., Klein D. (2000). The case of the missing pitch templates: how harmonic templates emerge in the early auditory system. J. Acoust. Soc. Am..

[bib79] Siebert W.M. (1970). Frequency discrimination in auditory system - place or periodicity mechanisms?. Proc. IEEE.

[bib1a] Shera C.A., Guinan J.J., Oxenham A.J. (2002). Revised estimates of human cochlear tuning from otoacoustic and behavioral measurements. Proc. Nat. Acad. Sci..

[bib80] Suga N., O'Neill W.E., Manabe T. (1978). Cortical neurons sensitive to combinations of information-bearing elements of biosonar signals in the mustache bat. Science.

[bib81] Sumner C.J., Palmer A.R. (2012). Auditory nerve fibre responses in the ferret. Eur. J. Neurosci..

[bib82] Sumner C.J., Wells T.T., Bergevin C., Sollini J., Kreft H.A., Palmer A.R., Oxenham A.J., Shera C.A. (2018). Mammalian behavior and physiology converge to confirm sharper cochlear tuning in humans. Proc. Natl. Acad. Sci. U.S.A..

[bib83] Taberner A.M., Liberman M.C. (2005). Response properties of single auditory nerve fibers in the mouse. J. Neurophysiol..

[bib84] Teich M.C., Khanna S.M., Guiney P.C. (1993). Spectral characteristics and synchrony in primary auditory-nerve fibers in response to pure-tone acoustic stimuli. J. Stat. Phys..

[bib85] Temchin A.N., Recio-Spinoso A., van Dijk P., Ruggero M.A. (2005). Wiener kernels of chinchilla auditory-nerve fibers: verification using responses to tones, clicks, and noise and comparison with basilar-membrane vibrations. J. Neurophysiol..

[bib86] Trahiotis C., Stern R.M. (1994). Across-frequency interaction in lateralization of complex binaural stimuli. J. Acoust. Soc. Am..

[bib87] Verschooten E., Desloovere C., Joris P.X. (2018). High-resolution frequency tuning but not temporal coding in the human cochlea. PLoS Biol..

[bib88] Verschooten E., Joris P.X. (2014). Estimation of neural phase locking from stimulus-evoked potentials. J. Assoc. Res. Otolaryngol..

[bib89] Verschooten E., Robles L., Joris P.X. (2015). Assessment of the limits of neural phase-locking using mass potentials. J. Neurosci..

[bib90] Wakeford O.S., Robinson D.E. (1974). Lateralization of tonal stimuli by the cat. J. Acoust. Soc. Am..

[bib91] Weiss T.F., Rose C. (1988). A comparison of synchronization filters in different auditory receptor organs. Hear. Res..

[bib92] Weiss T.F., Rose C. (1988). Stages of degradation of timing information in the cochlea: a comparison of hair-cell and nerve-fiber responses in the alligator lizard. Hear. Res..

[bib93] Wever E.G. (1949). Theory of Hearing.

[bib94] Winter I.M., Palmer A.R. (1990). Responses of single units in the anteroventral cochlear nucleus of the Guinea pig. Hear. Res..

[bib95] Wier C.C., Jesteadt W., Green D.M. (1977). Frequency discrimination as a function of frequency and sensation level. J. Acoust. Soc. Am..

[bib96] Young E.D., Oertel D., Shepherd G.M. (2004). Cochlear nucleus. The Synaptic Organization of the Brain.

[bib97] Zhang X., Heinz M.G., Bruce I.C., Carney L.H. (2001). A phenomenological model for the responses of auditory-nerve fibers: I. Nonlinear tuning with compression and suppression. J. Acoust. Soc. Am..

[bib98] Zook J.M., DiCaprio R.A., Thomas J.A., Kastelein R.A. (1990). A potential system of delay lines in the dolphin auditory brainstem. Sensory Abilities of Cetaceans: Laboratory and Field Evidence, NATO ASI Series.

[bib99] Zwicker E. (1952). Die Grenzen der Hörbarkeit der Amplitudenmodulation und der Frequenzmodulation eines Tones. Acustica.

[bib100] Zwicker E. (1956). Die elementaren Grundlagen zur Bestimmung der Informationskapazität des Gehörs (The foundations for determining the information capacity of the auditory system). Acustica.

